# Bond Behavior of Steel Cords Embedded in Inorganic Mortars

**DOI:** 10.3390/ma15155125

**Published:** 2022-07-23

**Authors:** Francesca Roscini, Maurizio Guadagnini

**Affiliations:** Department of Civil and Structural Engineering, The University of Sheffield, Sheffield S1 3JD, UK; m.guadagnini@sheffield.ac.uk

**Keywords:** SRG (steel reinforced grout), ductility, bond behavior, pull-out, cohesive numerical model, non-linear finite element

## Abstract

This paper investigates the bond behavior of steel cords embedded in inorganic matrices. A series of pull-out tests were carried out on individual galvanized steel cords embedded in either a cementitious or lime-based mortar matrix and the corresponding bond-slip relationships were derived. The quality of bond between cord and mortar was found to be critically affected by the workability of the mortar and its ability to create adequate composite action along the entire embedment length of the cord. The more workable lime-based mortar was found to guarantee a better interaction with the steel cord, in terms of initial bond stiffness, maximum bond strength, and post-peak behavior. The experimentally derived bond–slip relationships were subsequently integrated in a 3D non-linear finite element framework and used to determine the constitutive relationship of a surface-based cohesive contact between cord and mortar. The cohesive bond behavior was used to conduct a series of parametric studies on cords embedded in a lime-based mortar and examine the stress development within specimens with cords of different embedment lengths and subjected to different loading conditions (i.e., pull-out and direct tension). The active ‘Stress Transfer Zone’ was found to be about 125 mm, while an ‘Effective Transfer Radius’ of approximately 3.5–4 mm was identified. The numerical investigation implemented in this paper enabled one to study key interaction properties of steel reinforced grouts and can assist the design of more effective strengthening solutions.

## 1. Introduction

Over the past two decades, TRM (Textile Reinforced Mortar) systems have been increasingly used for the repair and strengthening of concrete [1,2,3,4] and masonry structures [5,6,7] as they can improve significantly structural performance without substantially altering geometry, masses and stiffness. The superior performance of this innovative solution based on textiles embedded within inorganic matrices has been demonstrated in several research documents available in the literature [8,9]. In addition, the use of TRM can ensure that preservation criteria are successfully met when strengthening architectural heritage [10,11]. Among the different types of textiles that can be used as main reinforcing material in TRM, steel textiles have been shown to provide exceptional properties in terms of both strength and ductility [12,13], as a result of the efficient bond that can develop at the fiber-to-mortar interface [14,15]. The performance of Steel Textile Reinforced Mortars (or grouts SRG) is influenced by the architecture (such as geometry and density) and the mechanical characteristics of the reinforcement, as well as the properties of the mortar, including grain size and mix design [16,17,18], number of reinforcement layers and presence of overlaps [19,20,21]. 

Much of the existing literature has focused on examining the bond performance of SRG systems to a masonry [22,23,24] or concrete substrate [25,26]. However, although the quality of bond between the textile and the mortar can critically affect the composite behavior of elements strengthened with these systems in terms of stiffness, ultimate capacity and failure mode [16,27,28], only limited work has investigated the interaction at the fiber-to-mortar interface [29,30,31]. This study addresses this research gap and examines the bond developed between steel cords and the surrounding inorganic mortar matrix at the mesoscale, as to gain invaluable insights into the intrinsic properties of SRG systems. 

The load–slip behavior of single galvanized Ultra High Tensile Strength Steel (UHTSS) cords embedded in two different types of mortar, cementitious and lime-based, is examined experimentally through a series of pull-out tests. Subsequently, the contact interface between cord and mortar is simulated in a non-linear finite element model implementing a surface-based cohesive behavior framework. The experimental results are used to calibrate the governing parameters of the cohesive law and define the initial linear elastic behavior prior to damage as well as the progressive degradation of the cohesive stiffness at the onset of debonding. For the SRG system with a lime-based mortar, for which the experimental tests enabled one to identify clearly the different stages of bond behavior, including adhesion, onset of debonding, damage initiation, maximum bond strength and post-peak bond degradation, the calibrated numerical model is then extended to perform a series of parametric analyses and examine the bond stress development along cords of different embedment lengths. The integrated experimental/numerical framework implemented in this study can be used to examine and determine the key interaction properties of textile reinforced mortars based on simple pull-out tests. This framework can be extended to include different fiber/matrix combinations and optimize the design of textiles and FRCMs to satisfy specific performance criteria, for example by defining minimum cord/layer spacing, or fiber to mortar strength ratio.

Finally, the framework illustrated in this paper can assist in the development of generalized bond–slip models that can be used for the design of more effective strengthening solutions. 

## 2. Experimental Program

A series of pull-out tests were performed on individual steel cords embedded in cementitious and lime-based mortar cubes (50 × 50 × 50 mm) to examine their bond–slip behavior [32]. The mechanical properties of the materials were characterized as described below. 

### 2.1. Materials Properties

Both the steel cords and the inorganic matrices used in this study were selected as representative of strengthening systems currently available on the market. The galvanized UHTSS cords (herein referred to as ‘HW’) are made of 2 wires twisted around 3 straight filaments (each with a nominal diameter of 0.37 mm) and have a total cross-sectional area of 0.538 mm^2^. The two inorganic mortars include a cementitious (GLT) and a lime-based mortar (GCF). The ‘GLT’ mortar is a fiber reinforced cement-based mortar with a crystalline reaction geo-binder base, while the ‘GCF’ specimens comprise a fine-grain mineral geo-mortar made from pure natural NHL range and geo-binder.

#### 2.1.1. Direct Tensile Tests on the Single Steel Cord

**Figure 1:**
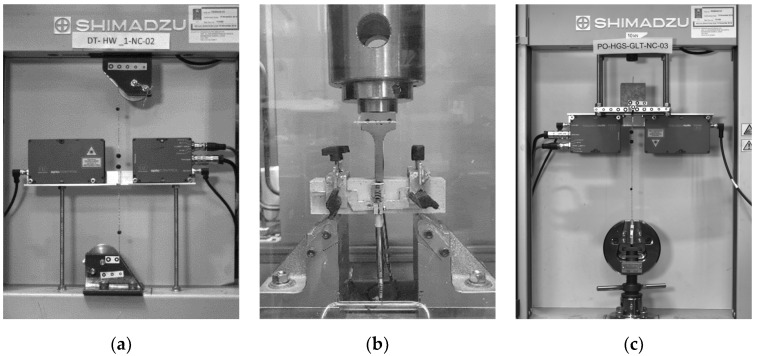
Experimental tests set-ups. (**a**) Direct tensile test on single UHTSS cord; (**b**) Flexural test on mortar prism; (**c**) Pull-out test on UHTSS cord embedded in mortar.

A bespoke setup comprising radiused clamps [33] using guide rollers with a relatively large radius was used to determine the main mechanical characteristics (Young’s modulus, failure stress and the corresponding strain) of the UHTSS cords. This clamping system was introduced to avoid stress concentrations in the gripping area and eliminate the need for bonded tabs (Figure 1a) [34]. A combination of contactless measuring methods was employed to monitor the elongation of the specimens, along with the measurements provided by the testing machine. Small spherical markers were crimped to the cord specimens at different locations and their movements were tracked using a laser LVDT and a high-definition camera, which was triggered at a frequency appropriate for the adopted test rate (1.2 mm/min) [24] under displacement control.

**Figure 2:**
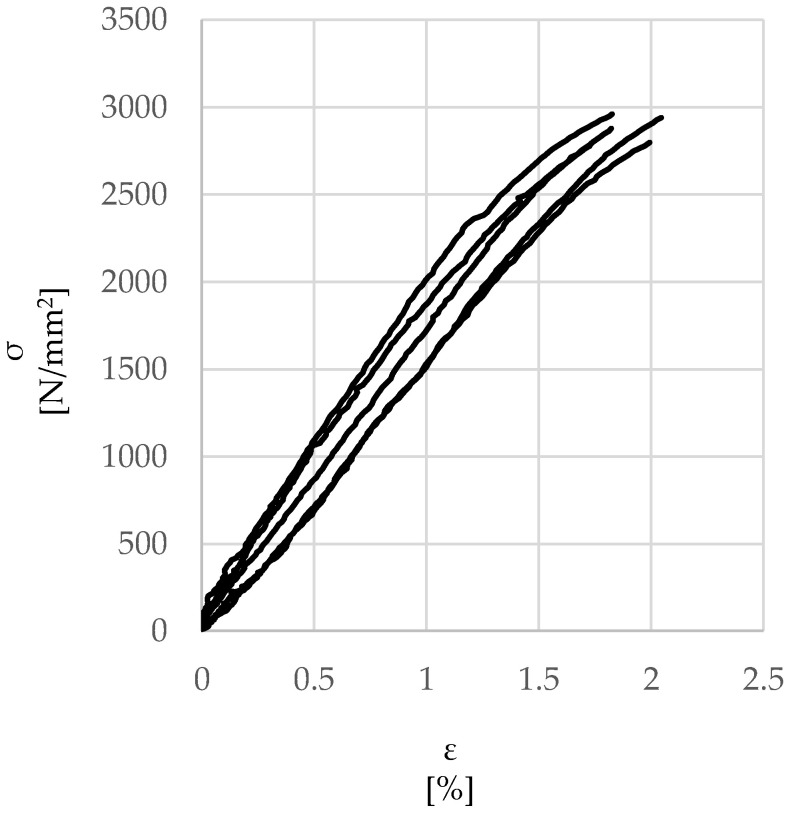
Stress–strain *(σ-ε)* response of UHTSS cords.

Figure 2 shows the tensile response obtained from the tests on 5 samples. The average direct tensile failure load was around 1700 N, corresponding to a maximum strength of approximately 3000 N/mm^2^ (COV = 2.3%), while the average strain at failure was approximately 1.8% (COV = 7.7%), thus yielding an average Young’s modulus of approximately 200 kN/mm^2^. The experimental values are aligned with those provided by the manufacturer.

#### 2.1.2. Flexural and Compressive Tests on Mortar Specimens

The mechanical properties of the mortars were characterized through a series of compressive and flexural tests, using a universal electromechanical testing machine, according to EN 1015-11 Annex B (2019) [35] at the testing time after 180 days from the casting. Three-point bending tests were carried out on 160 × 40 × 40 mm prisms (clear span of 100 mm) under displacement control at a rate of 0.5 mm/min, followed by compression tests on the resulting halves using bearing plates measuring 40 × 40 mm. A yoke equipped with two LVDTs (Figure 1b) was mounted on each of the prisms to measure the displacement at midspan and determine their flexural stiffness. The compressive tests were carried out under load control, imposing a 400 N/s rate for the ‘GLT’ cementitious mortar and a 200 N/s for the weaker ‘GCF’ lime-based mortar.

Table 1 summarizes the mechanical properties of the two mortars in terms of flexural strength (*f_t_*), stiffness (*E_t_*) and compressive strength (*f_c_*).

### 2.2. Pull-Out Tests

**Figure 3:**
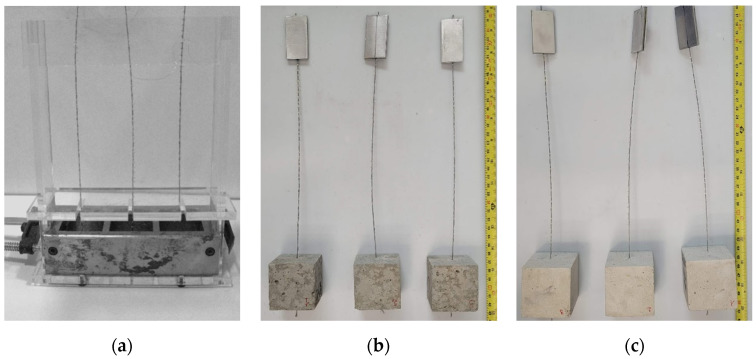
Manufacturing procedure for the pull-out tests. (**a**) Single cords positioned in a bespoke acrylic frame and steel mold. (**b**) ‘HW-GLT’ specimens equipped with tabs. (**c**) ‘HW-GCF’ specimens equipped with tabs.

**Figure 4:**
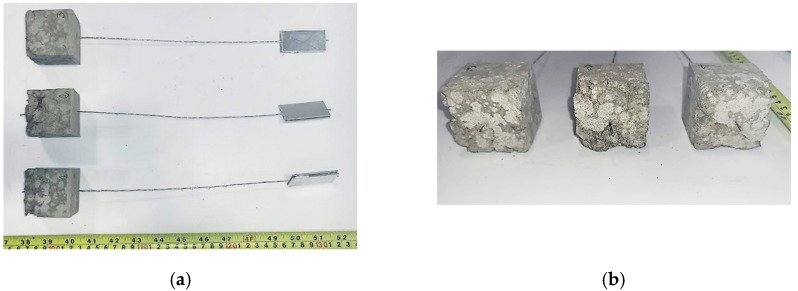
Presence of voids in ‘HW-GLT’ specimens as a result of the low workability of the cementitious mortar. (**a**) top view and (**b**) details of the mortar cubes.

The specimens for the pull-out tests were manufactured using a bespoke acrylic frame (Figure 3a). This was designed to hold the cords in position during casting and ensure that cord alignment was maintained during the entire curing period by fixing each cord to a crossbar. The steel cord was bonded for the entire cube height of 50 mm. The chosen bond length was deemed to be sufficiently short to provide information on local bond behavior, yet long enough to capture the effect of cord geometry (e.g., surface finish and twist angle). After demolding, the specimens were cured in a mist room (RH = 100%) to prevent shrinkage induced damage of the mortar matrix. It should be noted that, as reported in Figure 4a,b, the cementitious mortar of the ‘HW-GLT’ specimens was poorly compacted, owing to its low workability, and large voids were clearly identifiable along the embedded portion of the steel cord. As a result, a second casting was carried out for this system, and the mortar was vibrated to achieve a better compaction and mechanical interlock between the cord and the surrounding matrix. Before testing, the free end of the cord was glued within 50 mm-length tabs, resulting in a free length of around 250 mm between the tab and the mortar cube (Figure 3b,c).

**Figure 5:**
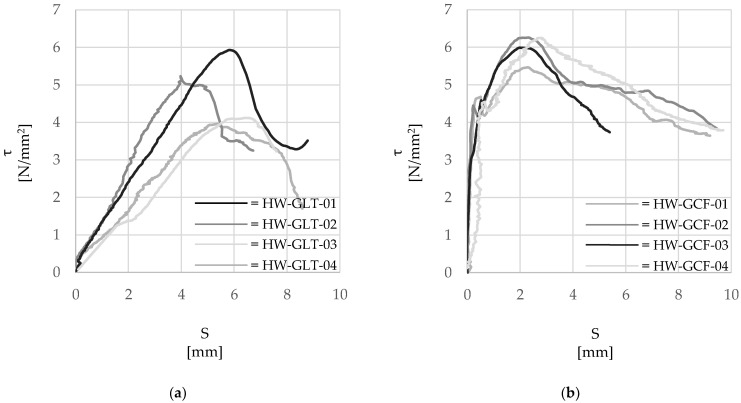
Experimental *τ*-slip (*τ-S*) response curves. (**a**) ‘HW-GLT’ system; (**b**) ‘HW-GCF’ system.

Subsequently, the pull-out tests were carried out using a universal testing machine equipped with a 10 kN load cell (Figure 1c), under displacement control at a loading rate of 0.3 mm/min according to Ghiassi et al. [36]. Two different measurement methods were used to obtain global and local deformations during the tests. The local elongation of the unbonded portion of the cord was detected by an optical laser LVDT system, while an optical ‘Point-Tracking’ method was used to measure the deformation of the system and the cord over different gauge lengths, as well as the relative displacement (slip) between the cord and the mortar cube. Uniquely identifiable markers were placed along the cord in the form of lead balls crimped directly onto the cord, while small adhesive circle markers were attached to the grips and the mortar cube (see Figure 1c). The experimental data were then processed in terms of τ-slip response curves, as reported in Figure 5a (‘HW-GLT’) and Figure 5b (‘HW-GCF’).

The average bond stress *‘τ’* is defined according to Equation (1). The slip of the reinforcement (*S*) relative to the mortar is determined by measuring the relative displacement between a marker placed on the mortar cube and a marker placed along the unbonded length of the cord (*δ*) and accounting for the elastic elongation of the portion of the cord between the reference marker and the surface of the mortar cube ($\varepsilon_{steel}$*d*), as shown in Equation (2).

(1) τ=FAcyl

where:

*τ* = average bond stress at the reinforcement-to-mortar surface (N/mm^2^).
*F* = Applied load (N).
*A_cyl_* = bonded surface of the cord (mm^2^).

(2) $$S=\delta -\varepsilon_{steel}\cdot d$$

where:

*δ* = displacement recorded between the un-bonded portion of the cord and the surface of the mortar cube (mm).
*ε_steel_* = steel strain detected by the laser method (%).
*d* = the distance between the reference marker and the surface of the mortar (mm).

### 2.3. Pull-Out Tests Results

Table 2 and Table 3 summarize the main results of the pull-out tests in terms of maximum applied load (*F_b_*), average maximum bond stress (*τ_max_*) and corresponding slip, exploitation ratio (*F_b_/F_u_*) and stiffness of the initial elastic response (Bond stiffness).

From the analysis of Figure 5, it is clear that the bond between the cord and the surrounding mortar is highly influenced by the properties of the mortar, with specimens HW-GLT developing on average smaller values of bond strength at higher slip.

#### 2.3.1. Steel Cord and Cementitious Mortar: ‘HW-GLT’ System

The bond stress–slip curves are characterized by a linear elastic branch up to the maximum bond stress of relatively low stiffness (on average equal to 120 N/mm), thus indicting that any significant contribution of chemical bond or mechanical interlock of the cord within the mortar did not develop. This was possibly due to the relatively low workability of the mortar and the poor contact with the cord created during the manufacture of the specimens. The exploitation ratio, defined as the ratio between the maximum axial stress developed in the cord and its tensile strength, was about 36%. Slip increased linearly with the applied load until the maximum strength was achieved, followed by a linear softening response and residual strength of friction [37]. 

#### 2.3.2. Steel Cord and Lime Based Mortar: ‘HW-GCF’ System

Conversely to specimens ‘HW-GLT’, the lime-based mortar seems to ensure a good degree of adhesion and mechanical interlock with the steel cord and improve stress distribution between the steel cord and mortar. As can be observed from Figure 5b, minimal slip (around 0.4 mm) is observed up to an average bond stress of about 4 N/mm^2^. This initial elastic phase is characterized by a relatively high stiffness (2440 N/mm), and it is followed by a nonlinear hardening stage up to a peak average bond strength of about 6 MPa and a more gradual softening stage. An exploitation ratio of up to 47% was found for the cords embedded in lime-based mortar, which is approximately 30% higher than that observed for cords embedded in cementitious mortar. This increase can be attributed to the better distribution of mortar along the length of the cord when using the more workable lime-base mortar. A residual average bond strength of about 4 MPa is still developed in these specimens at a relatively high slip of 8 mm. The higher initial stiffness developed by the ‘HW-GCF’ specimens can be attributed to the enhanced chemical adhesion activated at the cord-to-matrix interface and the high degree of penetration of the mortar within the indentations of the twisted filament, which is possibly promoted by the higher fines content. Overall, although similar values of bond strength can be developed between the steel cord and the two types of mortars investigated in this study, the use of a lime-based mortar results both in a higher degree of bond even at low slip levels and in a more ductile post-peak behavior.

## 3. Numerical Simulation 

Much of the existing literature on the numerical modelling of FRCMs investigates their behavior in pure tension [38,39,40] or focuses on examining the bond between FRCMs and the substrate [41,42,43]). The textile is considered to be fully bonded to the mortar [38] or their interaction is modelled using a cohesive interface [44] or explicitly using spring elements [43] and adopting various interface laws, including a linear, multi-linear or exponential softening branch (e.g., [28]). The interaction properties (textile to mortar or mortar to substrate) are typically calibrated using experimental data collected from either global slip measurements [28] or distributed strain measurements along the textile [27]. This study examines the bond developed between steel cords and the surrounding inorganic mortar matrix at the mesoscale, similarly to the work presented in [29], so as to gain invaluable insights into the intrinsic properties of SRG systems.

**Figure 6:**
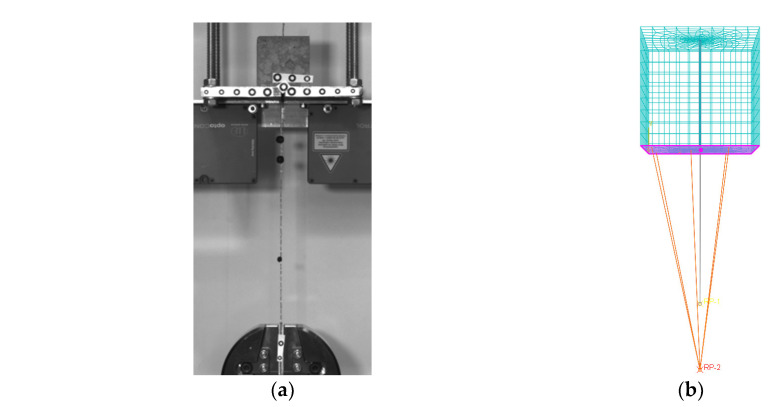
(**a**) Experimental set-up ‘HW-GLT’ system; (**b**) FE model and boundary conditions.

**Figure 7:**
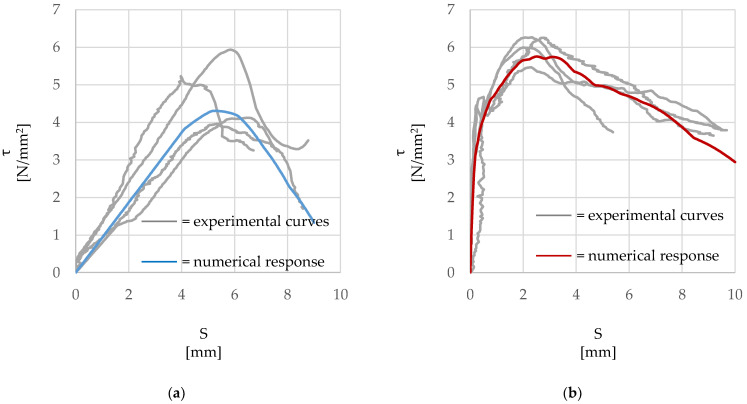
Numerical and experimental τ-slip (*τ-S*) response. (**a**) ‘HW-GLT’ system; (**b**) ‘HW-GCF’ system.

The experimental response curves provided above (Figure 5) were used to calibrate a proposed numerical model via a non-linear finite element analysis (Abaqus v6.14). The specimen was modelled with 3D elements and both the geometry and the materials properties were set as derived from the experimental tests. The boundary conditions were defined to simulate the experimental constraints and are shown in Figure 6. The surface of the cube in contact with the reaction frame was kinematically coupled to a reference point, the displacement of which was restrained in all directions, while a controlled displacement was imposed to the loaded-end of the cord. The bond behavior between the external surface of the steel cord and the surrounding mortar was simulated through the implementation of a cohesive ‘surface-to-surface’ contact interface. The behavior between surfaces is described through three steps: (1) before damage in terms of an elastic traction-separation response (cohesive behavior); (2) damage initiation; (3) a non-linear damaged interface response (damage evolution). The three stages implemented in the model are described in detail in the following sections, while the overall numerical responses are shown in Figure 7, along with the experimental data. 

### 3.1. The Cohesive Behavior

Initially, the mechanical contact property has been implemented as a cohesive behavior, and its governing parameters were defined using an inverse analysis approach. The traction response for separation through the interface was defined as ‘uncoupled’, as it is governed only by the principal directions. For this reason, the ‘*t*’ interfacial stresses at the surface depend on the elastic stiffness matrix *‘K’*. In particular, the *‘K_nn_, K_ss_, K_tt_’* diagonal values are the only properly calibrated from the experimental outcomes (see Figure 5). As a result, the *‘K_ns_, K_nt_, K_st_’* extra-diagonal ones are set to zero, as influenced by the shear behavior [45,46,47]. The Equation (3) reports the relation between the normal and the shear stresses to the normal and shear separations across the surface. In detail, *K* denotes the elastic stiffness matrix, the nominal traction stress vector is defined by *t*, where *t_n_*, *t_s_* and *t_t_* are respectively the normal and the two tangential local directions. Then, the relative separations are represented by *δ_n_*, *δ_s_*, and *δ_t_*.

(3) $$t=\left\{\begin{array}{c} t_{n}\\ t_{s}\\ t_{t} \end{array}\right\}=\left[\begin{array}{ccc} K_{nn} & K_{ns} & K_{nt}\\ K_{ns} & K_{ss} & K_{st}\\ K_{nt} & K_{st} & K_{tt} \end{array}\right]\left\{\begin{array}{c} \delta_{n}\\ \delta_{s}\\ \delta_{t} \end{array}\right\}=K\delta$$

### 3.2. Damage Evolution

The initiation of material degradation coincides with the end of the elastic stage and can be identified through a specific damage initiation criterion. For instance, the maximum nominal stress criterion was chosen in this simulation as described in Equation (4).

(4) $$\max\left\{\frac{t_{n}}{t_{n}^{0}},\frac{t_{s}}{t_{s}^{0}},\frac{t_{t}}{t_{t}^{0}}\right\}=1$$

The values of *t_s_* and *t_t_* at the onset of damage were taken as equal and derived from the experimental data, while a high value was chosen for *t_n_* to force damage only along the surface of the cord. The values of bond stress at the initiation of damage were taken as 4 MPa and 2 MPa for ‘HW-GLT’ and ‘HW-GCF’ specimens, respectively.

**Figure 8:**
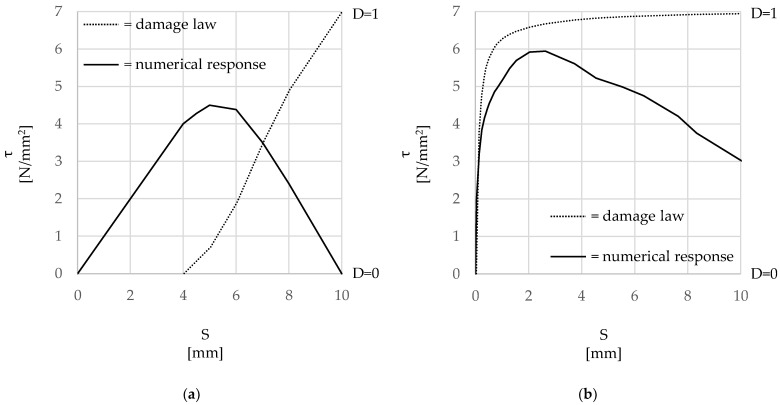
Damage laws proposed with the corresponding τ-slip (*τ-S*) numerical curve. (**a**) ‘HW-GLT’ system; (**b**) ‘HW-GCF’ system.

The rate of the cohesive stiffness degradation at the surface is governed by the variable *D*, which describes the shear stress damage at increasing levels of slip (Figure 8). The proposed damage laws are shown in Figure 8 along with the corresponding experimental τ-slip curves. Figure 8a shows the almost linear relationship adopted to simulate the bond damage evolution observed in the steel cords of the ‘HW-GLT’ specimens and the resulting bilinear τ-slip curve. Figure 8b shows the damage initiation and evolution behavior adopted for the ‘HW-GCF’ specimens along with the resulting numerical τ-slip curve. The exponential law used to model the bond between the steel cord and the lime-based matrix can capture more accurately the better adhesion and mechanical interlock observed experimentally, as well as the more gradual post-peak softening behavior. For simplicity, and given the relatively low stresses that were expected to develop within the mortar in both tension and compression, the mortar matrix was modelled as elastic material.

## 4. Parametric Study on Bond Length 

Amongst the various parameters affecting bond behavior [48,49], one of the most significant is the bonded length. For this reason, the cohesive law derived from the experimental tests on 50 mm-embedded length, which can be taken as representative of local bond behavior, has been adopted to simulate the pull-out performance at different lengths. The specific objective of this study is to provide an appropriate method to detect the effective bond length. This analysis has been implemented only to examine the performance of the ‘HW-GCF’ system, where the main stages of bond, including adhesion, mechanical interlock and damage evolution, are clearly identified [37].

### 4.1. Effective Bond Length

**Figure 9:**
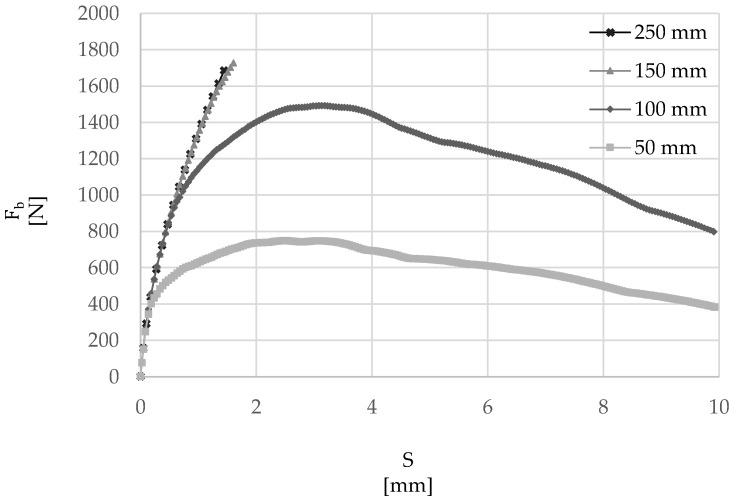
Load–Slip (*F_b_-S*) relationship, for cords with 50–100–150–250 mm embedded length.

**Figure 10:**
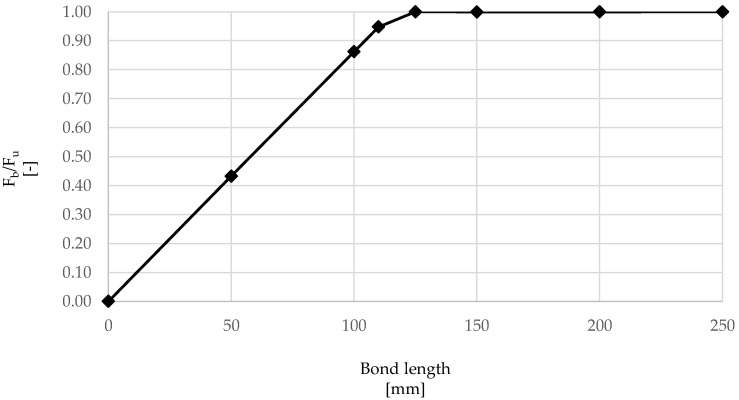
Variation of *F_b_/F_u_* with bond length.

The numerical model developed above was used to examine the effect of embedment length on overall maximum force at debonding (*F_b_*), average τ-slip behavior, failure mode and distribution of stresses along the cord–mortar interface. Firstly, the bond load–slip curves were examined (Figure 9), varying the embedment lengths from 50 to 250 mm. It can be seen that, while for values of 50 mm and 100 mm a peak bond force is achieved, followed by a softening branch, rupture of the cord occurred before the maximum bond strength was developed for 150 mm and 250 mm embedment lengths, thus indicating that the embedment lengths provided were greater that the effective bond length. As summarized in Table 4 and Figure 10, which include values of the exploitation ratio (FbFu), measured as the ratio between *F_b_* and the force at rupture of the cord *F_u_* (around 1700 N), the effective bond length was found to be approximately 125 mm. 

The results of this analysis are in line with previous studies on single cords [27,28] embedded in lime-based mortar, for which the values of effective lengths were found to be about 150–200 mm.

### 4.2. Stress Development

**Figure 11:**
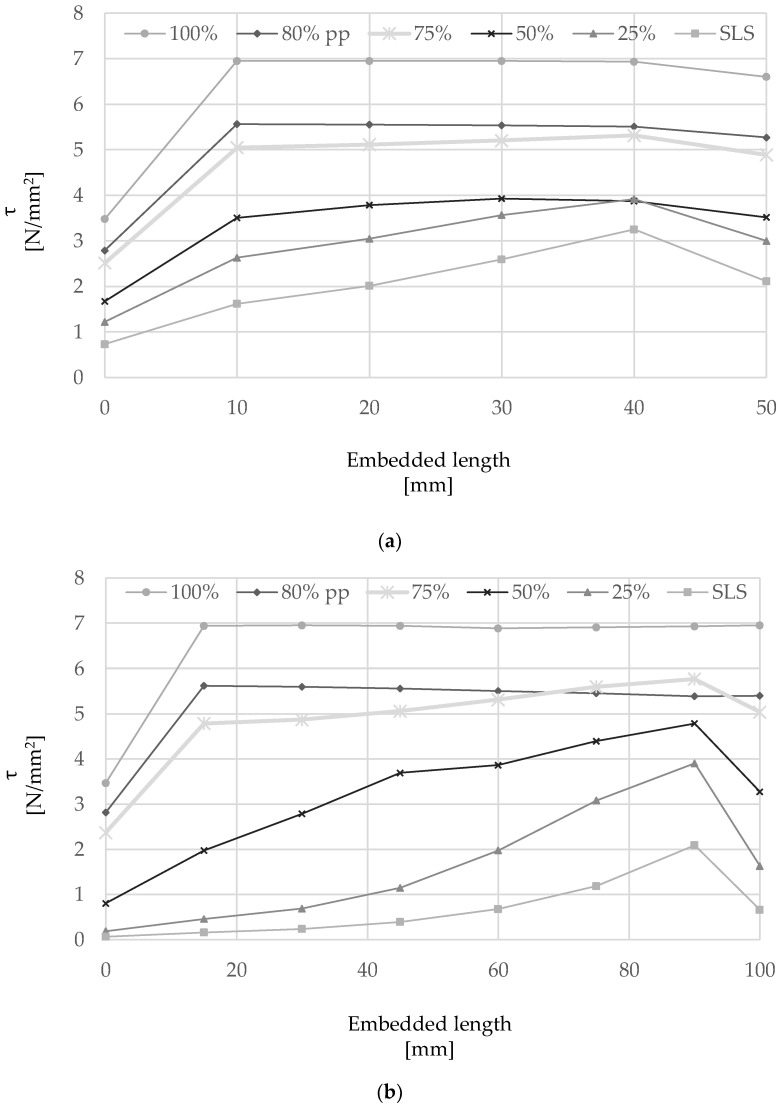

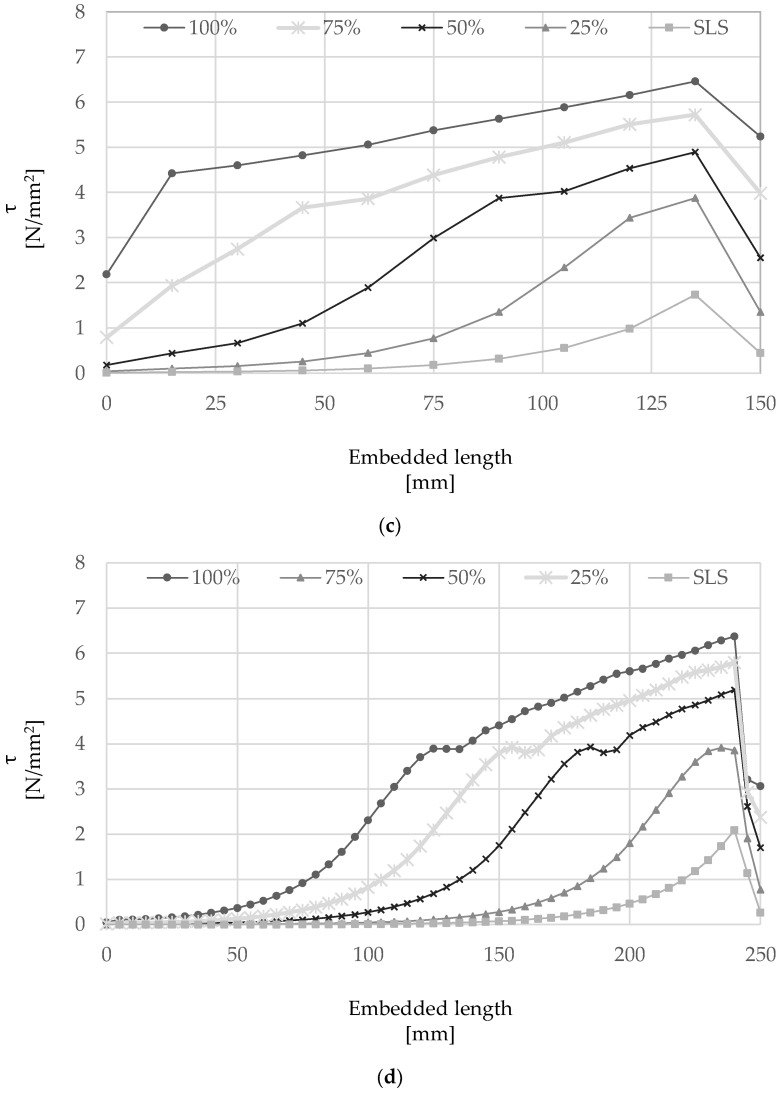
Shear stress distribution at various load levels along cords of different embedment lengths: (**a**) 50 mm, (**b**) 100 mm, (**c**) 150 mm and (**d**) 250 mm.

The distribution of shear stress *(τ)* along the single cord for selected embedment lengths, including 50 mm, 100 mm, 150 mm and 250 mm, is shown in Figure 11a–d at different performance levels: (i) Serviceability Limit States (‘SLS’); (ii) 25%, (iii) 50%, (iv) 75% and (v) 100% of the maximum pull-out load. When present, the shear stress distribution is also shown for a residual bond strength equal to 80% of the peak bond strength (‘80%pp’). The SLS performance level was taken assuming a service strain in the steel cord equal to 80% of the yield strain of conventional steel reinforcement (i.e., 0.16%) [50].

In general, higher values of shear stress are generated within a short distance from the loaded end and the distribution along the embedded length becomes more uniform as the pull-out load is increased. 

Specimens with an embedment length of 50 mm, as the ones tested experimentally, show that the variation between the peak bond stress and the average bond stress is relatively small, thus indicating that the selected embedment length is appropriate to characterize bond behavior at a local scale and define the properties of the cohesive contact implemented numerically. An increase in embedment length to 100 mm still enables the full development of the τ-slip curve, with the majority of the stress being transferred within the first 50 mm from the loaded end for loads up to approximately 50% of *F_b_* and reaching a maximum peak stress of approximately 7 N/mm^2^. An almost uniform stress distribution is shown for loads approaching *F_b_* as well as in the post-peak region, when the entire length of the cord is moving relatively to the surrounding mortar.

The maximum local bond stress mobilized in specimens with an embedment length greater than 150 mm was always smaller than 7 N/mm^2^ and rupture of the cord was always achieved before the maximum pull-out force could develop (see Section 4.1). From the analysis of the behavior of a cord with an embedment length of 250 mm (Figure 11d), it is clear that the majority of the force is transferred to the mortar within the first 125 mm from the loaded-end, identifying an active ‘Stress Transfer Zone’ [51] and an ‘ineffective length’.

**Figure 12:**
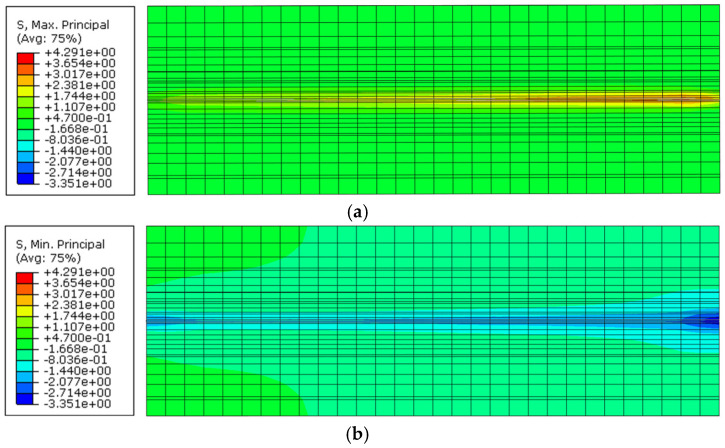
Principal stresses of the mortar: (**a**) maximum (tension) and (**b**) minimum (compression).

**Figure 13:**
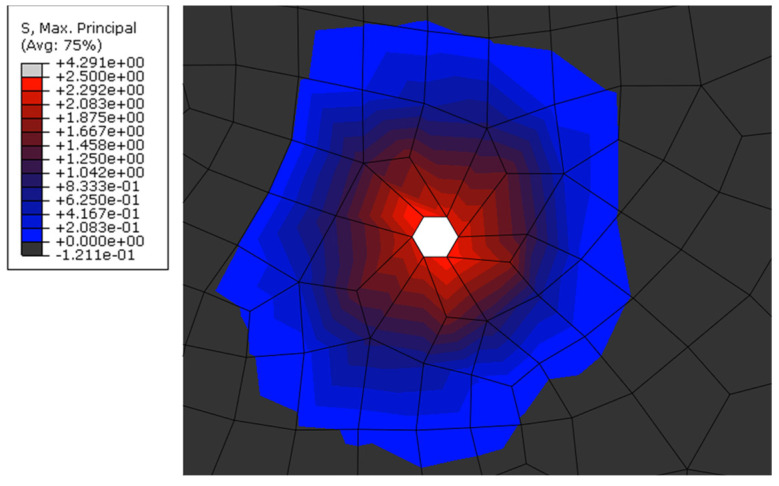
Maximum principal stresses of mortar around the single steel cord.

The distribution of principal stresses within a specimen was examined in more detail to investigate the influence of the loading arrangement (e.g., boundary conditions) on the overall bond behavior, as well as to assess the portion of mortar surrounding an embedded cord that can potentially be affected by a high stress state [52]. Figure 12a,b show the contour plots of maximum and minimal principal stresses, respectively, along a central cross-section of a mortar prism with a cord embedded for a length of 150 mm. As can be seen, the presence of the reaction plate causes a larger area of mortar around the cord and in the vicinity of the loaded-end to be subjected to compression, which in turn can provide additional confinement and affect the distribution of local bond stress, albeit only for a relatively short distance. The case with an embedded length of 150 mm was selected for this analysis as it enables the development of the full tensile capacity of the cord and the results from this analysis can be used to estimate the maximum radial distance from the cord at which critical tensile stresses are transferred. From the analysis of Figure 13, which shows the distribution of maximum principal stresses along a section perpendicular to the cord, it can be seen that the tensile force is transferred to the surrounding mortar over an ‘effective transfer radius’ of approximately 3.5–4 mm. It should be noted that only maximum principal stresses are shown in this figure to aid the analysis, and that the uniaxial tensile strength of the mortar was taken as 2.5 N/mm^2^ (estimated as approximately 50% of the experimental flexural strength, in line with existing provisions for concrete [53]). This information can be used to inform the optimal design of textiles and TRM systems, for example, in terms of spacing between cords, cord diameter, minimum mortar thickness and minimum distance between textile layers, and prevent premature failure modes in TRM-strengthened elements, such as delamination of mortar layers (e.g., ‘B’ and ‘C’ failure modes [54]) as a result of stress concentration between cords of high-density textiles.

## 5. Tension Stiffening Application

The damage law developed for the ‘HW-GCF’ system from simple pull-out tests has also been implemented to examine the bond stress distribution along elements subjected to direct tension. To this end, the behavior of a 400 mm long mortar prism reinforced by a single cord positioned in its center was modeled adopting the same strategy discussed in previous sections. It should be noted that, taking advantage of symmetry, only half of the specimen was model, for a length of 200 mm (greater than the established effective length of the cord under examination). The tensile response of the mortar was modelled using a bilinear curve, with the yield stress equal to the estimated uniaxial tensile strength (2.5 N/mm^2^).

**Figure 14:**
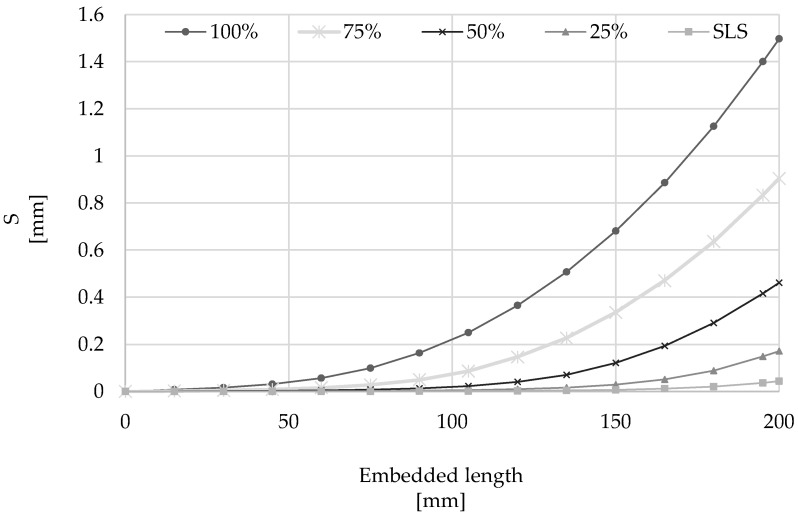
Distribution of slip along the embedded length of 200 mm at several load levels.

**Figure 15:**
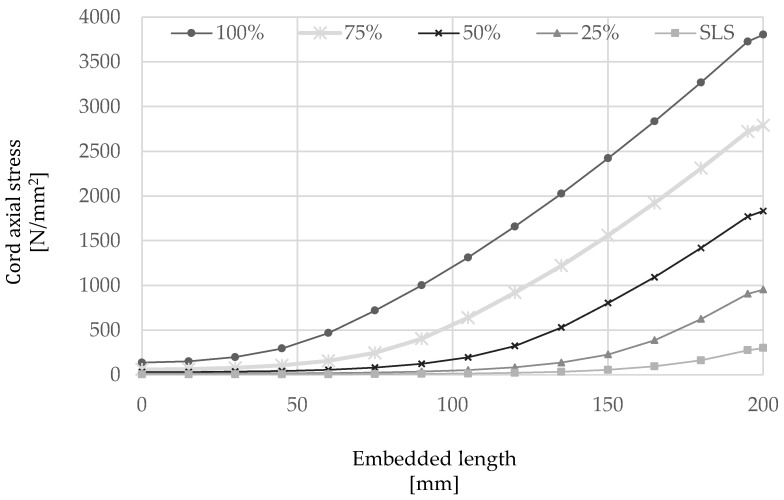
Distribution of cord axial stress along the embedded length of 200 mm at several load levels.

**Figure 16:**
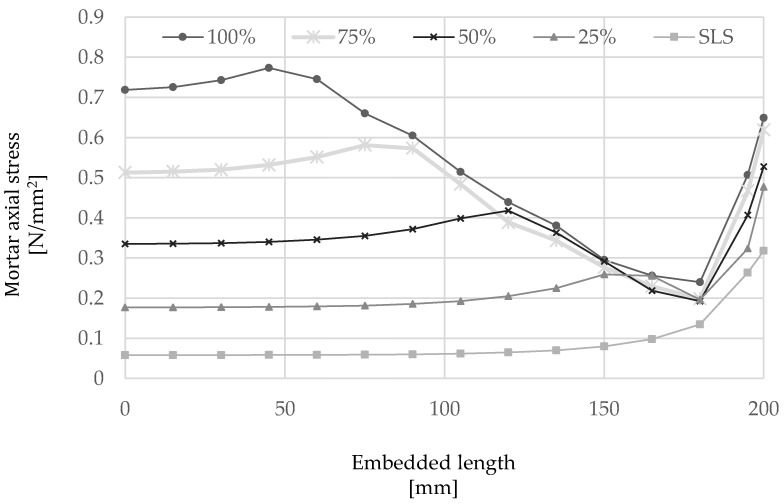
Distribution of axial stress in the mortar along the embedded length of 200 mm at several load levels.

The outcome of the numerical analysis is shown in Figure 14 and Figure 15 in terms of slip and stress along the cord, respectively, as well as the axial stress developed along the mortar interface (Figure 16). From the analysis of the results, it can be seen that slip occurs mainly along the first 150 mm of the cord from the loaded-end (the right-hand side of the plots), thus confirming the behavior observed from the analysis of elements subjected to direct pull-out, while only minimal slip is developed along an ‘ineffective length’ of approximately 50 mm about the middle of the element.

As shown in Figure 15, the axial stress along the cord decreases almost linearly moving away from the loaded-end and tends to zero towards the middle of the element. On the other hand, the axial stress in the mortar (Figure 16) is shown to decrease rapidly in the vicinity of the loaded-end, as a result of the slip induced within this region, followed by an almost linear increase up to a maximum value that is reached at a distance from the loaded end progressively higher as the applied load increases and is transferred from the cord to the surrounding mortar. As can be seen in Figure 16, the mortar axial stress was generally largely under its elastic limit.

## 6. Conclusions

The main aim of this study was to examine experimentally the bond behavior of individual cords embedded in a mortar matrix and develop a modeling strategy to determine the local τ-slip response through the implementation of an inverse analysis technique. Two different cohesive laws have been identified to describe the behavior of steel cords embedded in a cementitious and a lime-based mortar matrix. The validated model was then used for the case of steel cords embedded in a lime-based mortar to conduct a parametric study. Based on the experimental results discussed above and the outcome of the numerical analyses, the following conclusions can be drawn:

− The use of a mortar with low workability can result in poor compaction along the surface of the steel cord and an overall lower bond performance at considerably high levels of slip.
− The initial stiffness of the bond–slip response of cords embedded in cementitious mortar was found to be relatively low (120 N/mm).
− The use of the more workable lime-based mortar guarantees a better interaction with the steel cord and can lead to a considerably high initial bond stiffness (2440 N/mm) and a more ductile post-peak softening stage.
− A cohesive ‘surface-to-surface’ interaction formulation can be used to capture adequately the bond behavior of steel cords embedded in different types of mortars. The governing parameters (i.e., initial stiffness and damage initiation) as well as damage evolution can be calibrated based on experimental data obtained from simple pull-out tests.
− The effective bond length of the steel cords used in the ‘HW-GCF’ system was found to be approximately 125–150 mm.
− The analysis of the stress distribution in the matrix surrounding the steel cord revealed an effective stress transfer radius of approximately 3.5–4 mm.

The numerical approach implemented in this study, along with simple mechanical testing, can be used to examine the composite behavior of FRCM with different fiber/matrix combinations. The effect of other geometrical parameters, such as multiple cords and overlap regions, requires further investigation and should be included to enable a systematic optimization of FRCM, for example, in terms of minimum cord/layer spacing, or fiber-to-mortar strength ratio. The development of a suitable local bond stress–slip behavior will provide a more reliable estimate of the performance of FRCM, and provide the ability to model more complex loading scenarios and predict the effect of local failure modes on the overall performance of structures strengthened with these innovative systems.

## Figures and Tables

**Table 1:** Mechanical properties of the mortar matrices.

Mortar	*f_t_* (N/mm^2^)	*E_t_* (kN/mm^2^)	*f_c_* (N/mm^2^)
Cementitious (GLT)	9	13	36
Lime based (GCF)	5	2.8	19

**Table 2:** Experimental results for the ‘HW-GLT’ systems under pull-out tests.

HW-GLT	*F_b_* (N)	*τ_max_* (N/mm^2^)	Slip at *τ_max_* (mm)	*F_b_/F_u_*	Bond Stiffness ^1^ (N/mm)
01	535	4.1	6.5	0.31	71
02	771	5.9	5.8	0.45	139
03	680	5.2	4.2	0.40	176
04	515	4.0	5.3	0.30	101
Average	607	4.7	5.6	0.36	120

^1^ Secant stiffness of the elastic phase of the load-slip curve.

**Table 3:** Experimental results for the ‘HW-GCF’ systems under pull-out tests.

HW-GCF	*F_b_* (N)	*τ_max_* (N/mm^2^)	Slip at *τ_max_* (mm)	*F_b_/F_u_*	Bond Stiffness ^1^ (N/mm)
01	711	5.5	2.3	0.42	2168
02	814	6.3	2.3	0.48	2259
03	779	6.0	2.0	0.46	2931
04	812	6.2	2.8	0.48	2622
Average	795	6.1	2.3	0.47	2440

^1^ Secant stiffness of the elastic phase of the load–slip curve.

**Table 4:** Maximum force at debonding *(F_b_)* and exploitation ratio *(F_b_/F_u_)* for different bond lengths.

Bond Length (mm)	*F_b_* (N)	FbFu (-)
50	747	0.43
100	1491	0.86
110	1639	0.95
125	1729	1.00
150	1727	1.00
200	1728	1.00
250	1730	1.00

## Data Availability

The data presented in this study are available on request from the corresponding author.
